# Introduction of Methyl Groups at C2 and C6 Positions Enhances the Antiangiogenesis Activity of Curcumin

**DOI:** 10.1038/srep14205

**Published:** 2015-09-22

**Authors:** Hyun-Jung Koo, Sarah Shin, Joon Young Choi, Kyung-Han Lee, Byung-Tae Kim, Yearn Seong Choe

**Affiliations:** 1 Department of Nuclear Medicine, Samsung Medical Center, Sungkyunkwan University School of Medicine, Seoul, Korea; 2 Department of Health Sciences and Technology, SAIHST, Sungkyunkwan University, Seoul, Korea

## Abstract

Curcumin has diverse biological activities, but is known to undergo rapid metabolism via reduction of vinylic double bonds and phase II conjugation. To prevent reductive metabolism of curcumin, we introduced a methyl group at both C2 and C6 positions (compound **1**) or at the C2 position (compound **2**) of curcumin, creating steric hindrance on double bonds against metabolizing enzymes. As predicted, these compounds were resistant to reduction by alcohol dehydrogenase. Compound **1** was further evaluated for its antiangiogenesis activity *in vitro* and *in vivo*. It exhibited significantly greater inhibitory activity than curcumin against endothelial cell migration, invasion, and tube formation. Similarly, the *in vivo* Matrigel plug assay in C57BL/6 mice showed more pronounced reduction of blood vessels in the plugs containing **1** than those containing curcumin. Moreover, **1** suppressed tumor growth more effectively than curcumin in a U87MG mouse xenograft model by inhibiting angiogenesis. *In vivo* metabolite analysis by liquid chromatography/mass spectrometry demonstrated that **1** underwent markedly slower reductive metabolism than curcumin. Taken together, our results indicate that **1** has enhanced antiangiogenesis activity and suppression of tumor growth compared with curcumin, reflecting diminished reductive metabolism owing to the introduction of methyl groups at the C2 and C6 positions of curcumin.

5-Hydroxy-1,7-bis(4-hydroxy-3-methoxyphenyl)-1,4,6-heptatrien-3-one (curcumin), a major ingredient of the curry spice turmeric, has long been known for its anticancer, antioxidant, and antiinflammatory activities^1^,^2^,^3^. In particular, the anticancer activity of curcumin has been extensively studied to date. The mechanisms underlying the anticancer activity of curcumin have been shown to be diverse, including inhibition of cell proliferation and induction of apoptosis, and inhibition of transformation, tumor initiation, promotion, invasion, angiogenesis and metastasis^1^. Many studies have shown that curcumin has antiangiogenesis activity, which was demonstrated by the inhibition of endothelial cell proliferation and basic fibroblast growth factor (bFGF)-induced mouse corneal neovascularization^4^. This activity has been further corroborated by studies showing that curcumin inhibits FGF2-mediated gelatinase B expression and angiogenic responses in rabbit corneas^5^. In addition, curcumin has been found to inhibit tumor growth and angiogenesis in mice inoculated with human cancer cell lines, including glioma (U87MG) and ovarian cancer (SKOV3ip1 and HeyA8) cells^6^,^7^.

However, curcumin is severely susceptible to metabolism in rodents and humans and shown to undergo rapid metabolism in the liver and in the intestinal wall^1^,^8^,^9^,^10^,^11^. Studies to analyze and identify the metabolites of curcumin have been performed *in vitro* and *in vivo*. *In vitro* study has demonstrated that curcumin undergoes biotransformation into hexahydrocurcumin (HHC) and hexahydrocurcuminol when incubated with human or rat hepatocytes and analyzed using high performance liquid chromatography (HPLC) (280 nm) and mass spectrometry (MS), with more rapid metabolism observed in rat hepatocytes^12^. Another study has shown that curcumin incubated with intestinal and hepatic cytosols is metabolized to HHC, tetrahydrocurcumin (THC) and curcumin sulfate, although human and rat cytosols show differences in ability to metabolize curcumin to its reduction and conjugation products^13^. *In vivo* metabolism studies of curcumin in rodents have also been reported; these studies, using HPLC (prior to and after treatment of β-glucuronidase) and MS, have shown that i.p. administration of curcumin to mice (100 mg/kg) yields curcumin glucuronide, dihydrocurcumin glucuronide, THC glucuronide, and THC as major metabolites in plasma^14^,^15^. After intravenous (i.v.; 40 mg/kg) or oral (500 mg/kg) administration of curcumin to rats, HPLC analysis by UV absorption at 420 nm showed curcumin glucuronide and sulfate as the major metabolites in rat plasma. HPLC analysis by UV absorption at 280 nm did not provide useful information due to the presence of many endogenous molecules, however, MS using selected ion monitoring mode showed the peaks corresponding to HHC, hexahydrocurcuminol, and HHC glucuronide in small amounts^12^.

Although whether the anticancer activity of curcumin derives from the compound itself or its metabolites is not fully understood, a study has shown that curcumin inhibits 12-*O*-tetradecanoylphorbol-13-acetate-induced tumor promotion in a carcinogen-initiated mouse skin, whereas THC has lower activity^16^, suggesting that the activity may not be attributable to the reductive metabolite. Similarly, 4′,4″-dimethoxycurcumin, a curcumin derivative with limited metabolism in liver microsomal incubation system, exhibited greater potential than curcumin for inhibiting HCT116 human colon cancer cell proliferation and inducing apoptosis and for suppressing tumor growth in a MDA-MB 435S human breast cancer cell xenograft model^17^,^18^.

In this study, we designed and synthesized novel curcumin derivatives substituted with a methyl group for the hydrogen on both C2 and C6 positions or the C2 position only of the 5-hydroxy-1,4,6-heptatrien-3-one backbone. The underlying premise of these substitutions is that they may create steric hindrance against reductive metabolizing enzymes and thus prevent or retard reductive metabolism. The doubly substituted derivative (**1**) was further explored for its antiangiogenesis activity *in vitro* and for its antitumor activity as well as metabolism in a U87MG mouse xenograft model.

## Results

### Two curcumin derivatives substituted with a methyl group at both C2 and C6 positions and at the C2 position only were designed and synthesized

**Figure 1:**
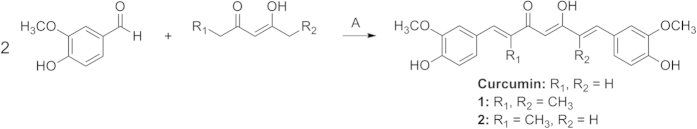
Synthesis of curcumin, 1, and 2. Reagents and conditions: (**A**) B_2_O_3_, ethyl acetate, (*n*-BuO)_3_B, *n*-BuNH_2_, 80 °C, 140 min; 1 N HCl, 50 °C, 30 min.

We synthesized two curcumin derivatives, one substituted with a methyl group for the hydrogen on both C2 and C6 positions (compound **1**) and a second derivative substituted with a methyl group for the hydrogen on the C2 position only (compound **2**) (Fig. 1). These compounds were designed to prevent reductive metabolism of curcumin, particularly to vinyl groups, by creating steric hindrance against metabolizing enzymes. Curcumin was also synthesized for comparison. Curcumin, **1**, and **2** were synthesized by aldol condensation of 1,3-diketone with two equivalents of vanillin in which the condensation was carried out through coupling of boron complex of the 1,3-diketone moiety with two equivalents of aldehyde in the presence of amine, followed by dilute acid treatment of the coupling product^19^,^20^. ^1^H NMR spectroscopic analyses showed that **1** existed as a 2.7:1 mixture of the enol and keto forms in DMSO-d_6_ at 25 °C, as confirmed by the difference in chemical shifts of proton(s) at C4 position (enol proton at 6.59 ppm and keto protons at 4.48 ppm)^21^. Additional ^1^H NMR spectroscopic experiments on **1** showed a 2.4:1 ratio of enol to keto form in DMSO-d_6_ at 40 °C, but higher ratios of enol to keto forms in acetone-d_6_ at 40 °C (6.2:1), 25 °C (7.3:1), and 0 °C (7.9:1). This result suggests that **1** may undergo facile tautomeric interconversion owing to the presence of methyl groups at the C2 and C6 positions of curcumin. In contrast, both **2** and curcumin existed solely as the enol form.

The *E,E*-configurations of the 2,6-dimethyl groups of **1** were confirmed by two-dimensional rotating-frame nuclear Overhauser effect spectroscopy (ROESY)^22^,^23^,^24^. These experiments showed no cross-peaks between methyl protons (H_a_) at C2 and C6 positions (2.15 ppm) and vinylic protons (H_b_) at C1 and C7 positions (7.61 ppm), indicating that these methyl and vinylic protons do not exist in close proximity in space (<5 Å). However, cross-peaks were observed between methyl protons H_a_ and two *ortho*-phenyl protons (7.04 ppm and 7.12 ppm) and between methyl protons H_a_ and a C4 proton (6.59 ppm). These data demonstrate that the dimethyl groups of **1** are in *E,E*-configurations (see Supplementary Fig. S1 online).

### Compounds 1 and 2 resisted reduction by alcohol dehydrogenase

**Figure 2:**
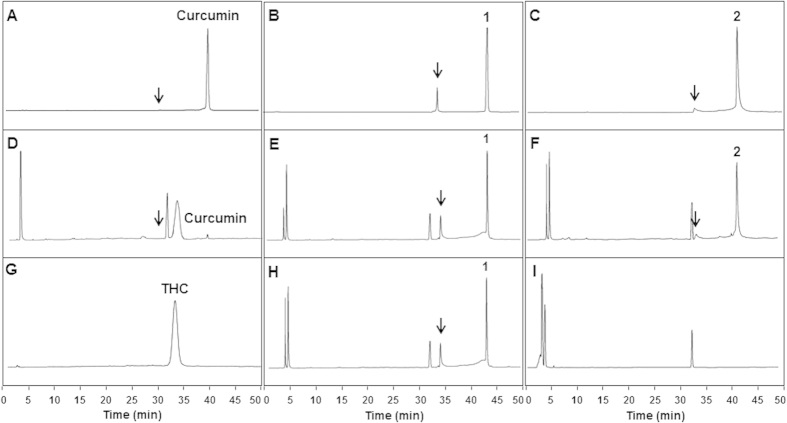
Reduction of curcumin, 1, and 2 by alcohol dehydrogenase. Incubation mixtures of compounds with alcohol dehydrogenase were analyzed by HPLC with UV detection at 280 nm. (**A**) Curcumin; (**B**) **1**; (**C**) **2**; (**D**) curcumin + alcohol dehydrogenase + NADPH; (**E**) **1** + alcohol dehydrogenase + NADPH; (**F**) **2** + alcohol dehydrogenase + NADPH; (**G**) THC standard; (**H**) **1** + alcohol dehydrogenase; (**I**) alcohol dehydrogenase. Arrows indicate the enol form of the corresponding compound.

Compounds **1** and **2** were incubated with alcohol dehydrogenase, which is known to be responsible for the reductive metabolism of curcumin^9^,^13^,^25^, and the results were compared with those of curcumin using HPLC (Fig. 2). Curcumin is known to exist as a keto-enol tautomer in which the keto form is favored under acidic or neutral conditions (pH 3–7)^8^,^26^. At the slightly acidic pH of HPLC solvents (pH 4.5), curcumin, **1**, and **2** appeared as two peaks on HPLC (Fig. 2A–C). Curcumin existed mostly as the keto form (t_R_ = 38.6–39.5 min), with less than 1% as the enol form (t_R_ = 28.8–29.7 min) (Fig. 2A), consistent with results reported in the literature^8^,^26^. Based on the retention times of curcumin, our data indicate that **1** exists as the keto form (t_R_ = 44.0–44.4 min) with 12% as the enol form (t_R_ = 33.8–34.2 min) (Fig. 2B), and **2** exists mostly as the keto form (t_R_ = 41.2–43.4 min) with 4% as the enol form (t_R_ = 32.8–33.8 min) (Fig. 2C). When the more non-polar peaks of **1** and **2** were collected and re-analyzed by HPLC under the same conditions, they eluted as two peaks with the same retention times and ratios as the first two peaks, indicating that they existed as keto-enol tautomeric mixtures (data not shown). Interestingly, curcumin appeared predominantly as the keto form; however, the enol form increased to 4% with introduction of a single methyl group (compound **2**) and 12% with introduction of two methyl groups (compound **1**) (Fig. 2A–C).

In control incubations omitting alcohol dehydrogenase, no peaks other than the compounds themselves were detected (data not shown). However, under conditions including alcohol dehydrogenase but omitting β-nicotinamide adenine dinucleotide 2′-phosphate (NADPH) and compound, a peak appeared at a retention time of 31.9–32.2 min (Fig. 2I), indicating the presence of an unknown impurity derived from alcohol dehydrogenase (Fig. 2D–F,H). The same peak was also detected in incubations omitting only NADPH (Fig. 2H for **1** and data not shown for both **2** and curcumin). Under complete incubation conditions (i.e., in the presence of alcohol dehydrogenase and NADPH), most curcumin (96%) was converted into THC, as indicated by a retention time (32.5–34.2 min) (Fig. 2D) that was indistinguishable from that for a THC standard (32.8–34.2 min) (Fig. 2G), whereas **1** and **2** remained intact without being reduced (Fig. 2E,F).

### Compound 1 exhibited cytotoxicity similar to curcumin

**Figure 3:**
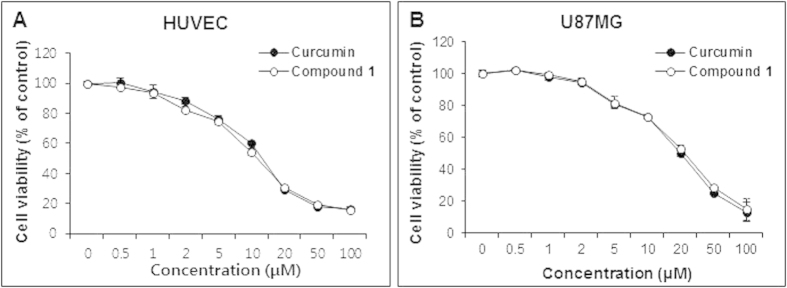
Cytotoxicity of curcumin and 1 in HUVECs and U87MG cells. Cell viability of (**A**) HUVECs and (**B**) U87MG cells was determined using MTT assays after treatment with curcumin or **1** for 72 h. Data are expressed as means ± S.E.M. (n = 3).

Cytotoxicity of **1** and curcumin was evaluated in human umbilical vein endothelial cells (HUVECs) and U87MG human glioblastoma cells using MTT assays. Cell viability of HUVECs after treatment of **1** and curcumin did not show any difference (Fig. 3A), and that of U87MG cells demonstrated a similar pattern to HUVECs (Fig. 3B).

### Compound 1 showed greater inhibitory activity than curcumin against endothelial cell migration, invasion, and tube formation *in vitro*

**Figure 4:**
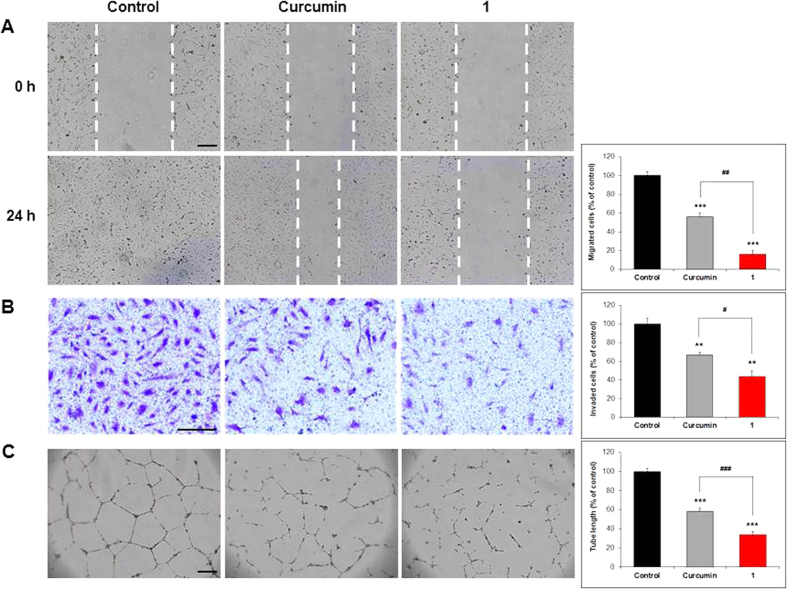
Inhibition of endothelial cell migration, invasion, and tube formation. (**A**) HUVEC migration in wound healing assays. Cell monolayers were wounded (white broken lines) and treated with vehicle (control), curcumin, or **1**. After 24 h, the migrated cells were photographed and quantified (bar graph). (**B**) HUVEC invasion in Transwell assays. Cells were seeded in the top chamber and treated with vehicle (control), curcumin, or **1**. After 24 h, the cells that had invaded through the membrane were stained, photographed, and quantified (bar graph). (**C**) Tube formation by HUVECs. Cells were cultured on Matrigel and treated with vehicle (control), curcumin, or **1**. After 16 h, tube-like structures were photographed and quantified (bar graph). Data are expressed as means ± S.E.M. (n = 3). **P < 0.01 and ***P < 0.001 vs. control. ^#^*P* < 0.05, ^##^*P* < 0.01, and ^###^*P* < 0.001 vs. curcumin. Scale bar, (**A**–**C**) 200 μm.

For wound healing migration, Transwell invasion and tube formation assays, HUVECs were treated with 10 μM curcumin or **1** to avoid excessive cytotoxicity to the cells. The ability of **1** to inhibit endothelial cell migration was measured using a wound healing migration assay. Cells treated with **1** maintained the full width of the wound gap 24 h after wounding, whereas those treated with vehicle showed full migration into the wounded area during the same time period (Fig. 4A). A quantitative analysis showed a marked difference in the migration ability of cells treated with **1** (15.9%) compared with curcumin (56.0%) (^##^*P* < 0.01); in both cases, migration ability was significantly lower than that for vehicle-treated controls (defined as 100%) (***P < 0.001) (Fig. 4A, bar graph). The invasive ability of endothelial cells was tested by assaying passage of cells through a Matrigel-coated membrane. Cells were treated with vehicle, curcumin or **1**, and cells that had invaded through the membrane were stained. The amount of invading cells was much less for cells treated with **1** than for those treated with curcumin or vehicle (Fig. 4B). A quantitative analysis revealed invasive values relative to controls (100%) of 43.8% for **1** and 66.5% for curcumin (**P < 0.01), indicating that, although both **1** and curcumin significantly inhibited endothelial cell invasion, **1** was more effective (^#^*P* < 0.05) (Fig. 4B, bar graph). Tube formation by endothelial cells, an *in vitro* measure of angiogenesis, was assessed after incubating cells with vehicle, curcumin, or **1** for 16 h. Photographs of cells showed that tube formation was inhibited more strongly by **1** than by curcumin (Fig. 4C). Compared with controls (100%), the average tube length for cells treated with **1** (33.9%) was markedly shorter than that for curcumin-treated cells (57.9%) (^###^*P* < 0.001), and both were significantly shorter than those in vehicle-treated control cells (***P < 0.001) (Fig. 4C, bar graph).

### Compound 1 inhibited endothelial cell proliferation through suppression of VEGFR2-mediated ERK1/2 signaling pathway

**Figure 5:**
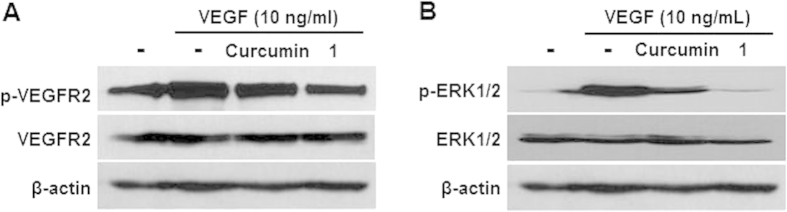
Inhibition of endothelial cell proliferation. HUVECs were pre-treated with curcumin or **1** and then stimulated with VEGF. Phosphorylation levels of (**A**) VEGFR2 and (**B**) ERK1/2 were analyzed by Western blotting. β-Actin was served as a loading control.

It has been shown that activation of VEGFR2 regulates endothelial cell proliferation through activation of the ERK1/2 signaling pathway^27^. Therefore, inhibitory activity of **1** on endothelial cell proliferation was confirmed by Western blot analysis using phospho-VEGFR2 and phospho-ERK1/2 antibodies. VEGF-induced phosphorylation of VEGFR2 and ERK1/2 was significantly suppressed in HUVECs treated with **1** compared with those treated with curcumin (Fig. 5A,B). The total protein levels of VEGFR2 and ERK1/2 were not affected by treatment of curcumin or compound **1**. These results suggest that compound **1** suppresses cell proliferation by inhibiting the ERK1/2 signaling pathway.

### *In vivo* mouse Matrigel plug assay showed reduced blood vessels in the plugs containing 1 compared with those containing curcumin

**Figure 6:**
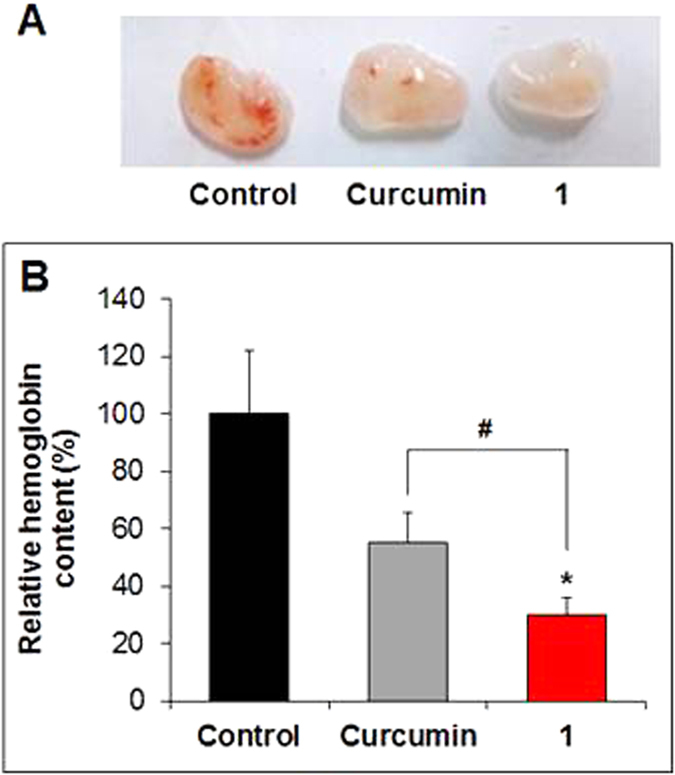
*In vivo* Matrigel plug assay. (**A**) Plugs obtained from mice 14 d after inoculation with Matrigel containing vehicle (control), curcumin, or **1**. (**B**) Relative hemoglobin content normalized to the weight of the corresponding plugs. Data are expressed as means ± S.E.M. (n = 4). *P < 0.05 vs. control. ^#^*P* < 0.05 vs. curcumin.

New blood vessel formation was investigated using Matrigel plug assays in C57BL/6 mice. Mice were inoculated with Matrigel containing bFGF and heparin plus vehicle, 10 μM curcumin, or **1**, and then plugs were analyzed after 14 d. Control plugs containing bFGF and heparin showed an abundance of red color, indicative of angiogenesis. In contrast, Matrigel plugs containing curcumin or **1** showed less or fainter color (Fig. 6A), as confirmed by an analysis of hemoglobin content in the plugs (Fig. 6B). The hemoglobin content normalized to the plug weight decreased to 30.0% for **1** and 55.2% for curcumin (^#^*P* < 0.05), relative to a value of 100% for controls (*P < 0.05) (Fig. 6B).

### Compound 1 inhibited tumor growth more effectively than curcumin in a U87MG mouse xenograft model

**Figure 7:**
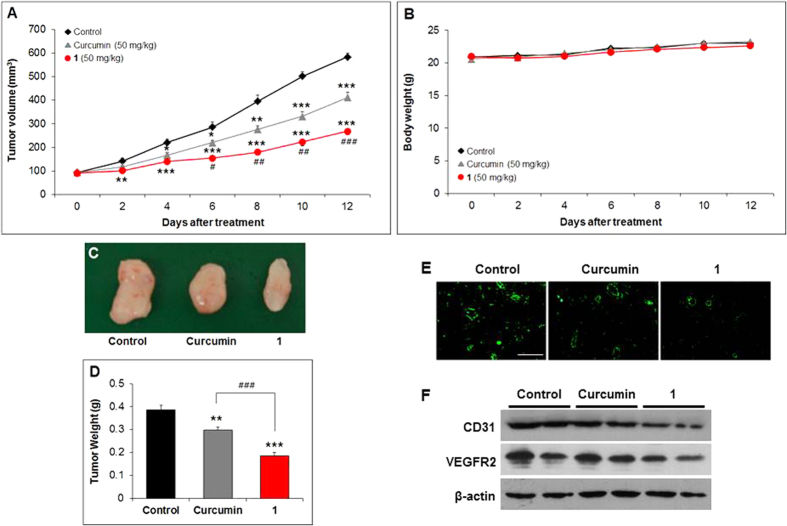
Inhibition of tumor growth. U87MG tumor-bearing mice were administered olive oil (control) (n = 8, black), curcumin (50 mg/kg, n = 8, grey), or **1** (50 mg/kg, n = 8, red) every other day for 13 days. (**A**) Tumor volumes and (**B**) body weights of mice were measured every other day during the same period. At the end of the experiment, excised tumor tissues were photographed (**C**) and weighed (**D**). Data are expressed as means ± S.E.M. *P < 0.05, **P < 0.01, and ***P < 0.001 vs. control. ^#^*P* < 0.05 , ^##^*P* < 0.01, and ^###^*P* < 0.001 vs. curcumin. (**E**) Immunofluorescence staining and (**F**) Western blotting of tumor tissues; U87MG tumor tissues were immunostained with anti-CD31 antibody and photographed (scale bar, 200 μm), and immunoblotted using anti-CD31 and anti-VEGFR2 antibodies.

The antitumor activity of **1** and curcumin was tested in a mouse xenograft model created by inoculating mice with U87MG cells. After treatment with vehicle (olive oil), curcumin, or **1**, mice were sacrificed and tumor tissues were excised. The tumor volume increased significantly in the control group, reaching an average volume of 584.52 ± 16.45 mm^3^. In contrast, growth was substantially suppressed in the treatment groups. Treatment with curcumin reduced the average tumor volume by 29.4% (to 412.67 ± 20.70 mm^3^) (***P < 0.001) whereas treatment with **1** reduced tumor volume by 54.2% (to 268.00 ± 15.53 mm^3^) (***P < 0.001) (Fig. 7A), results that translated into tumor growth-inhibition rates of 34.9% and 63.8% for curcumin and **1**, respectively. Moreover, tumor volumes of mice treated with **1** were significantly smaller than those treated with curcumin from day 4 of treatment onwards (^#^*P* < 0.05, ^##^*P* < 0.01, and ^###^*P* < 0.001) (Fig. 7A). There were no significant changes in body weights of mice during the course of treatment (Fig. 7B), indicating that the doses used were not toxic. Tumors extracted from the mice differed not only in size but also in the abundance of blood vessels, showing inhibition of blood vessel formation by **1** (Fig. 7C). Tumors removed from mice treated with curcumin and **1** weighed 0.297 ± 0.012 g and 0.187 ± 0.013 g, respectively–reductions of 23.1% and 51.6% relative to the control group (0.386 ± 0.020 g) (**P < 0.01, ***P < 0.001) (Fig. 7D).

### Immunofluorescence staining and Western blot analysis showed a significant inhibition of angiogenesis in tumor tissues from mice treated with 1

Immunofluorescence staining for the endothelial cell marker CD31 (cluster of differentiation 31) in tumor tissues extracted from mice treated with **1** showed decreased blood vessel density compared with those obtained from mice treated with curcumin or vehicle (Fig. 7E), suggesting that **1** suppressed tumor growth in a U87MG mouse xenograft model by inhibiting angiogenesis (Fig. 7A). Similarly, Western blot analyses showed that CD31 and vascular endothelial growth factor receptor 2 (VEGFR2) expression levels were lower in tumor tissues of mice treated with **1** than in those treated with curcumin or vehicle (Fig. 7F).

### Compounds 1 underwent diminished reductive metabolism *in vivo*

**Figure 8:**
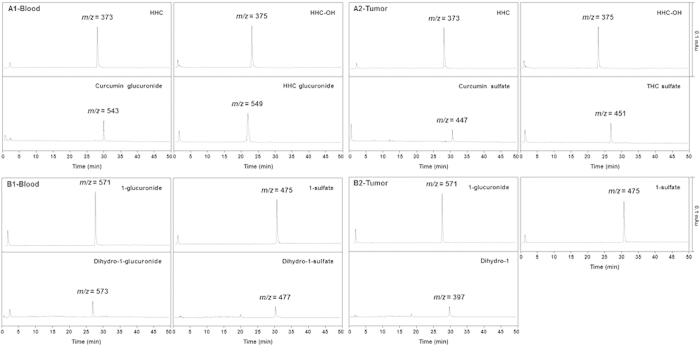
*In vivo* metabolism of curcumin and 1. U87MG tumor-bearing mice were injected i.p. with curcumin (50 mg/kg) or **1** (50 mg/kg). Samples of blood and tumor extracted from mice 30 min after injection of (**A**) curcumin or (**B**) **1** were analyzed by LC/MS using selected ion monitoring mode. (**A1**) Blood; (**A2**) tumor; (**B1**) blood; (**B2**) tumor. HHC: hexahydrocurcumin; HHC-OH: hexahydrocurcuminol; THC: tetrahydrocurcumin.

Samples of blood and tumor tissue from mice injected with curcumin or **1** were analyzed by LC/MS using selected ion monitoring mode. In mice injected with curcumin, reductive metabolites such as HHC (*m/z* = 373) and hexahydrocurcuminol (*m/z* = 375) were detected as the major metabolites in samples of blood and tumor tissue (Fig. 8A1). Small amounts of other metabolites were also detected: curcumin glucuronide (*m/z* = 543) and HHC glucuronide (*m/z* = 549) in the blood samples, and curcumin sulfate (*m/z* = 447) and THC sulfate (*m/z* = 451) in the tumor tissues. On the other hand, **1** was metabolized mostly to the conjugation products such as **1**-glucuronide (*m/z* = 571) and sulfate (*m/z* = 475) (Fig. 8B1). Dihydro-**1** (*m/z* = 397) and its conjugation products (*m/z* = 477 and 573) appeared as minor metabolites in the tumor tissue and blood samples, respectively. The peaks corresponding to curcumin (*m/z* = 367) and **1** (*m/z* = 395) were not detected.

## Discussion

It has been known that curcumin undergoes rapid metabolism *in vivo*, yielding its reductive metabolites, phase II conjugation products, and reductive metabolites with conjugation^1^,^8^,^9^,^14^. For the formation of these metabolites, the vinylic double bonds and phenolic OH groups of curcumin serve as the primary reaction sites^8^,^14^. In this study, we introduced a methyl group at both C2 and C6 positions or the C2 position of curcumin in order to prevent reductive metabolism (Fig. 1).

It has been reported that reductive metabolism on vinylic double bonds of curcumin is carried out by enzymes including alcohol dehydrogenase rather than by hepatic microsomes^9^,^13^,^25^. *In vitro* alcohol dehydrogenase-mediated reduction experiments demonstrated that most curcumin was transformed into THC (Fig. 2D), whereas **1** and **2** remained intact (Fig. 2E,F), which indicated that even introduction of a single methyl group at the C2 position was sufficient to prevent reduction.

Curcumin has been shown to have anticancer activity by modulating diverse molecular targets; downregulation of expression of growth factors, suppression of activation of transcription factors, inhibition of protein kinases, induction of cell cycle, induction of apoptosis, and others^1^,^28^. Compound **1** may also interact with these diverse molecular targets, like curcumin. Studies have shown that curcumin inhibits angiogenesis^4^,^5^,^6^,^7^, the process of new blood vessel formation that has an essential role in tumor growth and metastasis^29^. In this study, we found that the *in vitro* antiangiogenesis activity of **1** is significantly greater than that of curcumin against endothelial cell migration, invasion, and tube formation, which are crucial steps in the formation of new blood vessels during angiogenesis (Fig. 4)^30^. However, **1** has cytotoxicity similar to curcumin in HUVECs and U87MG cells, indicating that the antiangiogenesis activity of **1** may not be due to its cytotoxicity (Fig. 3). As an effort to elucidate the molecular targets of **1** for its antiangiogenesis activity, we carried out western blot analysis using phospho-VEGFR2 and phospho-ERK1/2 antibodies, because ERK1/2 phosphorylation plays a critical role in endothelial cell proliferation^27^. The results showed that **1** inhibited endothelial cell proliferation more significantly than curcumin at the concentrations (10 μM) used for the *in vitro* antiangiogenesis activity assays (Fig. 5). These *in vitro* study data suggest that the antiangiogenesis activity of **1** may be at least in part attributable to the suppression of VEGFR2-mediated ERK1/2 signaling pathway.

Matrigel plug assays performed in C57BL/6 mice also verified the antiangiogenesis activity of **1** *in vivo* (Figs 4 and 5), indicating that the introduction of methyl groups at both C2 and C6 positions of curcumin enhances its *in vivo* antiangiogenesis activity. It has been also reported that curcumin inhibited tumor growth and angiogenesis in tumor mouse xenograft models; curcumin reduced tumor volume by 68% in a U87MG xenograft model 25 days after i.p. administration (60 mg/kg) compared with controls and inhibited tumor-induced angiogenesis^6^. In this study, we examined inhibition of tumor growth by **1** in a U87MG cell xenograft model of glioblastoma, in which angiogenesis is a pathological characteristic^31^,^32^. Compound **1** reduced tumor volumes and weights by 35.1% and 37.0%, respectively, compared with curcumin and by 54.2% and 51.6%, respectively, compared with vehicle (Fig. 7A,D). In our result, the extent of reduction in mean tumor volume of curcumin-treated mice compared with control mice is less than that reported in the literature^6^, which may be explained by differences in the doses used and duration of treatment. It should be also noted that the curcumin used for this study was more than 98% pure by HPLC; by comparison, commercially available curcumin contains ~17% demethoxycurcumin and ~3% bisdemethoxycurcumin. Consistent with the interpretation that the tumor growth inhibition reflects a decrease in the formation of new blood vessels, both immunofluorescence (Fig. 7E) and Western blot analyses (Fig. 7F) showed a decrease in the endothelial cell markers, CD31 and VEGFR2^33^,^34^, in tumor tissues from mice treated with **1** or curcumin compared with controls, with **1** having a greater effect than curcumin.

Metabolites extracted from mice injected with curcumin and **1** were analyzed by LC/MS using selected ion monitoring mode because of the presence of a host of endogenous molecules^12^. Curcumin was shown to undergo biotransformation into curcumin glucuronide, reductive metabolites with conjugation, and THC in plasma of mice 1 h after i.p. administration^14^. Similar to this result, our data showed that the metabolites of curcumin in the blood and tumor of mice were mostly derived from reductive metabolism (Fig. 8A1). Unlike curcumin, compound **1** was mostly metabolized to its glucuronide and sulfate with small amounts of dihydro-**1** and its conjugation products (Fig. 8B1). This result indicated that introduction of a methyl group at both C2 and C6 positions of curcumin significantly diminished reductive metabolism, because the reduction on one double bond to form dihydro-**1** is the first step of reductive metabolism^12^,^14^. In addition, it was shown that curcumin may undergo distinct metabolism, depending on the route of administration and species^12^,^14^. Oral administration of curcumin to rats provided curcumin glucuronide and sulfate as major metabolites with small amounts of reductive metabolites^12^, whereas i.p. administration to mice yielded curcumin glucuronide and reductive metabolites as major metabolites^14^. Moreover, curcumin glucuronide and sulfate were detected in plasma of healthy humans after oral administration^35^. Therefore, further studies are warranted to investigate whether oral or i.v. administration of **1** shows differences in metabolite formation.

## Conclusions

In this study, curcumin derivatives substituted with a methyl group at both C2 and C6 positions (**1**) or at the C2 position only (**2**) were synthesized with the goal of preventing the reductive metabolism of curcumin. *In vitro* studies showed that both **1** and **2** resisted reduction by alcohol dehydrogenase, whereas curcumin was mostly reduced to THC. Similarly, compound **1** underwent markedly limited reductive metabolism in mice, whereas curcumin underwent biotransformation mostly into reductive metabolites. Further evaluation of **1** to assess the effect of methyl groups on antiangiogenesis activity showed significantly greater inhibitory activity against endothelial cell proliferation, migration, invasion and tube formation compared with curcumin. Moreover, **1** inhibited tumor growth in a U87MG mouse xenograft model by inhibiting the formation of new blood vessels, which was more effective than treatment with curcumin. Collectively, our findings indicate that introduction of methyl groups at both C2 and C6 positions of curcumin enhances the antiangiogenesis activity and tumor growth inhibition compared with curcumin, possibly by diminishing reductive metabolism. To the best of our knowledge, this is the first study to design, synthesize, and evaluate curcumin derivatives capable of escaping reductive metabolism. The results of this study will provide a useful platform for the development of curcumin derivatives with enhanced antitumor activity.

## Methods

### Materials and equipment

All NMR samples were prepared in DMSO-*d*_*6*_ (20 mM). ^1^H NMR spectra were obtained at 25 °C using a Bruker Avance 500 (500 MHz) spectrometer (Rheinstetten, Germany), and chemical shifts (δ) were reported as the ppm downfield of the internal tetramethylsilane. Two-dimensional ROESY experiments were carried out using a mixing time of 700 ms and the states-TPPI mode on a Bruker Avance 600 (600 MHz) spectrometer. Fast atom bombardment mass spectra were obtained using a JMS-700 Mstation (JEOL Ltd, Tokyo, Japan).

### Synthesis of 1, 2, and curcumin

B_2_O_3_ (384.3 mg, 5.52 mmol) was added to an ethyl acetate solution (2.7 mL) of 3,5-heptanedione (634.2 μL, 4.60 mmol), which was then stirred at 80 °C for 40 min. To this, an ethyl acetate solution (2.7 mL) of vanillin (1.6 g, 10.58 mmol) and (*n*-BuO)_3_B (3.0 mL, 11.04 mmol) was added and stirred at 80 °C for 40 min. After slow addition of *n*-butylamine (0.36 mL, 3.68 mmol) at room temperature, the reaction mixture was stirred at 80 °C for 1 h and then treated with 1 N HCl (4.5 mL) at 50 °C for 30 min. At the end of the reaction, the mixture was treated with a saturated NaHCO_3_ solution, which was then extracted with ethyl acetate, washed with water, and dried over Na_2_SO_4_. Flash column chromatography (2:1 hexanes-ethyl acetate) followed by recrystallization from ethanol gave **1** (920 mg, 50%) as a yellow-colored solid. The ^1^H NMR spectrum showed a 2.7:1 mixture of enol and keto forms. **1** (see Supplementary Fig. S2 and Fig. S3 online): Purity (HPLC): 97.6%; ^1^H NMR (500 MHz, DMSO-*d*_*6*_): enol form, δ 2.15 (s, 6H), 3.81 (s, 6H), 6.59 (s, 1H), 6.86 (d, *J* = 8.0 Hz, 2H), 7.04 (dd, *J* = 8.0 and 1.5 Hz, 2H), 7.12 (d, *J* = 1.5 Hz, 2H), 7.61 (s, 2H); keto form, *δ* 2.02 (s, 6H), 3.80 (s, 6H), 4.48 (s, 2H), 6.87 (d, *J* = 8.0 Hz, 2H), 7.06 (dd, *J* = 9.0 and 1.5 Hz, 2H), 7.13 (d, *J* = 1.5 Hz, 2H), 7.62 (s, 2H); HRMS (*m/z*): [M + H]^+^ calcd. for C_23_H_25_O_6_, 397.1651; found, 397.1656. Configurations of 2,6-dimethyl groups of **1** were determined by 2D ROESY (see Supplementary Fig. S1 online).

Compound **2** and curcumin were synthesized according to the procedure described above using 2,4-hexanedione and 2,4-pentanedione, respectively. Flash column chromatography followed by recrystallization from ethanol gave **2** (850 mg, 42%) or curcumin (713 mg, 42%) as an orange-colored solid. The ^1^H NMR spectra of **2** and curcumin showed only the enol forms.

**2** (see Supplementary Fig. S4 and Fig. S5 online): Purity (HPLC): 99.5%; ^1^H NMR (500 MHz, DMSO-*d*_*6*_) δ 2.13 (s, 3H), 3.81 (s, 3H), 3.84 (s, 3H), 6.40 (s, 1H), 6.80 (d, *J* = 16.0 Hz, 1H), 6.82 (d, *J* = 8.0 Hz, 1H), 6.86 (d, *J* = 8.0 Hz, 1H), 7.04 (dd, *J* = 10.0 and 2.0 Hz, 1H), 7.12 (dd, *J* = 8.0 and 1.5 Hz, 1H), 7.13 (d, *J* = 1.5 Hz, 1H), 7.31 (d, *J* = 2.0 Hz, 1H), 7.53 (d, *J* = 15.5 Hz, 1H), 7.60 (s, 1H); HRMS (*m/z*): [M + H]^+^ calcd. for C_22_H_23_O_6_, 383.1495; found, 383.1503.

Curcumin (see Supplementary Fig. S6 and Fig. S7 online): Purity (HPLC): 98.8%; ^1^H NMR (500 MHz, DMSO-*d*_*6*_) δ 3.83 (s, 6H), 6.06 (s, 1H), 6.74 (d, *J* = 15.5 Hz, 2H), 6.82 (d, *J* = 8.0 Hz, 2H), 7.14 (dd, *J* = 8.0 and 1.5 Hz, 2H), 7.31 (d, *J* = 1.5 Hz, 2H), 7.54 (d, *J* = 16 Hz, 2H); HRMS (*m/z*): [M + H]^+^ calcd. for C_21_H_21_O_6_, 369.1338; found, 369.1338.

### Reduction with alcohol dehydrogenase

Reduction of compounds by alcohol dehydrogenase was tested according to a reported method with slight modifications^13^,^25^. Briefly, curcumin, **1**, or **2** was dissolved in 0.1% DMSO in phosphate buffer (0.01 M, pH 7.4), and the solution was pre-incubated with equine alcohol dehydrogenase (5 units/mL) at 37 °C for 3 min; the reaction was initiated by adding NADPH (5 mM) at the same temperature. The final volume of the incubation mixture was 0.5 mL. After 1 h, the incubation mixture was treated with acetate buffer (1 M, pH 4.5) and then extracted with a 9:1 mixture of ethyl acetate and 2-propanol. The organic layer was filtered, dried, and re-dissolved in HPLC solvents, and then analyzed by HPLC using a C18 column (YMC, 4.6 × 250 mm, 5 μm) with sequential gradients of 1% ammonium acetate-acetonitrile containing 0.05% acetic acid (pH 4.5). The first gradient program was from a 95:5 mixture to a 55:45 mixture over 30 min and the second gradient was from a 55:45 mixture to a 5:95 mixture over 20 min. The flow rate was 1 mL/min, and the eluent was monitored at 280 nm using a UV detector. The metabolite standard, THC, was synthesized as previously reported^25^,^36^.

### Cell lines and culture

HUVECs and U87MG cells were purchased from American Type Culture Collection (Manassas, VA, USA). HUVECs (passage 4–6) were maintained in endothelial cell growth medium-2 (EGM-2) (Lonza, Walkersville, MD, USA), and U87MG cells were cultured in Dulbecco’s modified Eagle medium (DMEM) (Gibco, Brooklyn, NY, USA) supplemented with 10% fetal bovine serum (FBS) (Gibco), streptomycin (100 μg/mL), and penicillin (100 units/mL). All cell lines were maintained at 37 °C in a humidified 5% CO_2_ incubator.

### MTT assay

HUVECs or U87MG cells were plated onto 96-well plates at a density of 1 × 10^4^ cells/well and incubated for 24 h to allow the cells to attach. Cells were treated with curcumin or compound **1** dissolved in DMSO for 72 h at different concentrations (0.5, 1, 2, 5, 10, 20, 50, and 100 μM). Final concentration of DMSO was 0.1%. After treatment, 40 μL of 3-(4,5-dimethylthiazol-2-yl)-2,5-diphenyltetrazolium bromide (MTT, Sigma-Aldrich) solution (2 mg/mL in PBS) was added to each well and incubated for 4 h at 37 °C. The resulting MTT formazan was dissolved in 100 μL of 0.04 N HCl/2-propanol, and the absorbance at 570 nm was measured using an ELISA microplate reader (Molecular Devices, Sunnyvale, CA, USA). All experiments were performed in triplicate.

### Wound healing migration assay

HUVECs were seeded in a 48-well plate at a density of 2 × 10^4^ cells/well in EGM2 medium and cultured to confluence. Wounds were generated by scraping a cell monolayer with a 200 μL pipette tip, after which cells were washed twice with PBS to remove cell debris. Cells were incubated at 37 °C for 24 h with EGM2 medium containing vehicle (DMSO), curcumin (10 μM), or **1** (10 μM). Images of wounds were obtained using an Olympus microscope and photographed. The migration distance of cells during the 24-h wound healing period was measured using the ImageJ program, available from http://imagej.nih.gov. Cell migration ability was calculated as follows: [(average distance between wounds at 0 h–average distance between wounds at 24 h)/average distance between wounds at 0 h] × 100. All experiments were performed in triplicate.

### Transwell invasion assay

Transwell membranes were coated with 50 μL of Matrigel (3× diluted; BD Biosciences, San Jose, CA, USA). After polymerization of the Matrigel at 37 °C for 30 min, HUVECs (1 × 10^5^ cells) suspended in 100 μL of serum-free medium containing vehicle (DMSO), curcumin (10 μM), or **1** (10 μM) were seeded onto the upper chamber. The lower chamber was filled with EGM2 medium. After incubation at 37^ ^ °C for 24 h, the residual cells in the Matrigel were removed using cotton swabs. The membrane was then fixed with methanol for 10 min, and cells that had invaded to the lower side of the membrane were stained with 0.05% crystal violet (Sigma-Aldrich, St. Louis, MO, USA). The stained cells in each well were photographed and the dye was released with 10% acetic acid. Absorbance of the solution was measured at 595 nm using a microplate reader (Molecular Devices, Sunnyvale, CA, USA). All experiments were performed in triplicate.

### Tube formation assay

Matrigel (50 μL/well) was added to the wells in a 96-well plate and allowed to polymerize at 37 °C for 30 min. HUVECs (10^4^ cells) treated with vehicle (DMSO) or 10 μM curcumin or **1** for 30 min were added to the top of the Matrigel. After incubation for 16 h, tube formation was assessed by photographing plates. Endothelial cell tube length was measured at ten random positions using the ImageJ program.

### Animal studies

All animal experiments were performed in accordance with the National Institutes of Health Guide for the Care and Use of Laboratory Animals and were approved by the Institutional Animal Care and Use Committee (IACUC) of Samsung Medical Center. Mice were maintained under specific pathogen-free conditions during the entire period of the studies.

### *In vivo* Matrigel plug assay

C57BL/6 mice (male, 7-week old) were divided into three groups: control (DMSO), curcumin, and **1**. Each compound (10 μM) dissolved in DMSO was mixed with Matrigel containing recombinant mouse bFGF (100 ng/mL) and heparin (50 units/ml), and then 0.4-mL aliquots were subcutaneously injected into the ventral regions of mice (n = 4 mice/group). After 14 d, mice were sacrificed, and the Matrigel plugs were removed, weighed, and homogenized in cold PBS. The hemoglobin content of the plugs was measured using Drabkin’s reagent kit 525 (Sigma-Aldrich) and normalized to the weights of the plugs.

### Tumor growth inhibition

U87MG cells (5 × 10^6^ cells per mouse) were subcutaneously inoculated into the right hind legs of 6-week-old BALB/c nude mice (male, n = 24). When tumor size had reached 90–100 mm^3^ (20–21 g, 1 week after inoculation), the mice were randomly divided into three groups (n = 8/group): vehicle control (olive oil), curcumin treated (50 mg/kg), and **1** treated (50 mg/kg). Mice in each group were injected i.p. with vehicle or compound every other day for 13 days. Tumor volumes and body weights of mice were measured every other day throughout the treatment period. Tumor volume was measured using a vernier caliper and was calculated from the following equation: (length x width^2^)/2^37^. Tumor growth inhibition rate (%) was calculated based on the equation, (CV–TV)/CV × 100, where CV was obtained by subtracting tumor volume in the control group measured on the first day of vehicle injection from that on the last day of injection, and TV was obtained by subtracting tumor volume in the treatment group measured on the first day of compound injection from that on the last day of injection^38^. On day 19, mice were sacrificed, and tumor tissues were excised and weighed.

### Immunofluorescence staining

Tumor tissues were fixed in 4% paraformaldehyde, embedded in paraffin, cut at 5 μm thickness, and applied to slides. Slide-mounted sections were deparaffinized in xylene and rehydrated in decreasing concentrations of ethanol. Thereafter, antigen retrieval was performed by placing slides in proteinase K (Dako Japan Inc., Tokyo, Japan). The slides were washed three times in deionized water for 2 min each, blocked by incubating with 5% normal goat serum for 30 min, incubated with anti-CD31 primary antibody (Abcam, Cambridge, MA, USA) for 2 h, and then incubated with a fluorescein isothiocyanate-conjugated secondary antibody (Santa Cruz Biotechnology, Santa Cruz, CA, USA) for 1 h. The slides were analyzed and photographed using a fluorescence microscope.

### Western blot analysis

HUVECs were grown to confluence in 100-mm dishes and serum-starved for 12 h. Cells were pre-treated 1 h with vehicle (DMSO), curcumin (10 μM), or **1** (10 μM) at 37 °C and then stimulated with VEGF (10 ng/mL) for 10 min. Equal amounts of protein in cell extracts were separated by sodium dodecyl sulfate-polyacrylamide gel electrophoresis (SDS-PAGE) on 10% gels and then transferred to polyvinylidene difluoride membranes (Millipore, Billerica, MA, USA). After blocking with 3% bovine serum albumin (w/v) in TBST (0.05 M Tris, 0.15 M NaCl, pH 7.5, and 0.1% Tween 20) at room temperature for 1 h, the membranes were immunoblotted using anti-phospho-ERK1/2, anti-ERK1/2, phospho-VEGFR2 (Cell Signaling Technology, Beverly, MA, USA), anti-VEGFR2, and anti-β-actin (Santa Cruz Biotechnology) antibodies, and then incubated with a horseradish peroxidase-conjugated secondary antibody. Immunoreactivity was detected using the Western Blotting Plus Chemiluminescence reagent (Thermo Scientific, Rockford, IL, USA). For tumor tissues, proteins were extracted by homogenizing the tissues. SDS***-***PAGE analysis and immunoblotting were performed as described above using anti-CD31 and anti-VEGFR2 antibodies.

### *In vivo* metabolism of compound 1 in U87MG tumor-bearing mice

U87MG cells were inoculated into the right hind legs of 6-week-old BALB/c nude mice (male). When tumor size had reached 340–350 mm^3^, the mice were injected i.p. with curcumin or compound **1** (50 mg/kg) dissolved in olive oil. At 30 min after injection, mice were sacrificed, and samples of blood and tumor were obtained, homogenized in 1 mL of ethanol, and centrifuged. The supernatants were diluted with HPLC solvents, passed through a Waters MassPREP on-line desalting cartridge (Milford, MA, USA), and then analyzed by LC/MS on a LCQ system (Thermo Finnigan, San Jose, CA, USA) in negative ion mode. LC was performed using a HPLC column (Unison US-C18, 2.0 × 150 mm, 5 μm; Imtakt, Portland, OR, USA) and conditions used for analysis of the *in vitro* incubation mixtures of **1** with alcohol dehydrogenase, and MS analysis was carried out using selected ion monitoring mode.

### Statistical analysis

Data are expressed as means ± S.E.M. Data were analyzed using one-way analysis of variance (ANOVA) followed by Tukey’s test using GraphPad software. Differences at the 95% confidence level (*P* < 0.05) were considered significant.

## Additional Information

**How to cite this article**: Koo, H.-J. *et al.* Introduction of Methyl Groups at C2 and C6 Positions Enhances the Antiangiogenesis Activity of Curcumin. *Sci. Rep.* **5**, 14205; doi: 10.1038/srep14205 (2015).

## Supplementary Material

Supplementary Information
