# Supplementary material for: Introduction of Methyl Groups at C2 and C6 Positions Enhances the Antiangiogenesis Activity of Curcumin
Source: Sci Rep. 2015 Sep 22;5:14205. doi: 10.1038/srep14205 (PMC4585748; doi:10.1038/srep14205)
Supplement: Supplementary Information [file srep14205-s1.doc]

**Supplementary Information**

**Introduction of Methyl Groups at C2 and C6 Positions Enhances the Antiangiogenesis Activity of Curcumin**

Hyun-Jung Koo^1*^, Sarah Shin^2*^, Joon Young Choi^1^, Kyung-Han Lee^1,2^, Byung-Tae Kim^1^ & Yearn Seong Choe^1,2^

^1^Department of Nuclear Medicine, Samsung Medical Center, Sungkyunkwan University School of Medicine, Seoul, Korea, ^2^Department of Health Sciences and Technology, SAIHST, Sungkyunkwan University, Seoul, Korea.


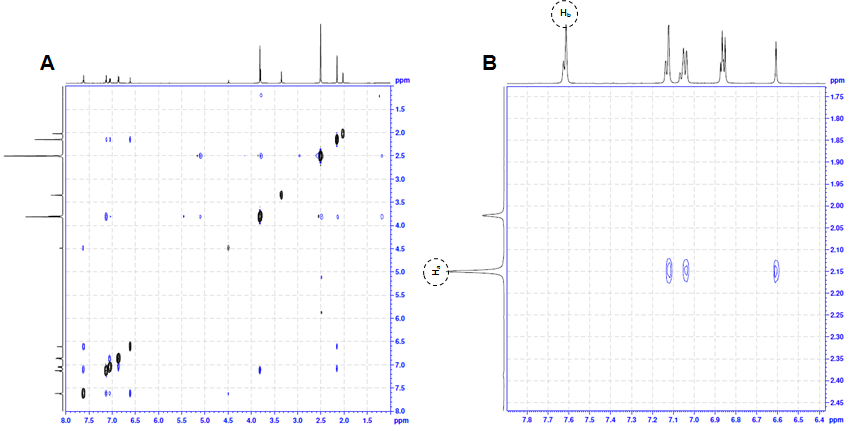


**Supplementary Figure 1.** **2D ROSEY spectra of compound 1.** A, full spectrum. B, partial enlargement of spectrum in (A). Methyl groups at C2 and C6 positions of **1** are confirmed to have *E,E*-configurations, as no cross-peaks were detected between methyl protons at C2 and C6 positions, indicated as H_a_ (2.15 ppm), and vinylic protons at C1 and C7 positions, indicated as H_b_ (7.61 ppm).


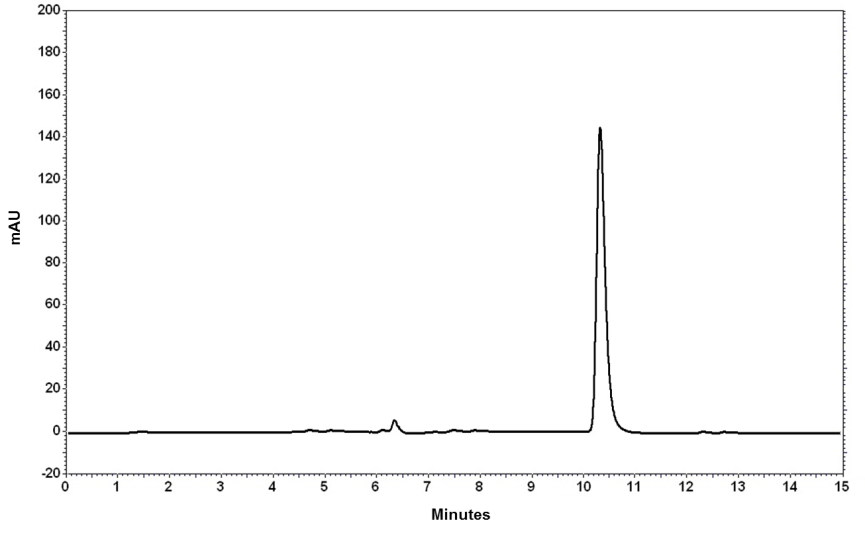


**Supplementary Figure 2. HPLC chromatogram of compound 1.** Compound **1** was analyzed by HPLC using a C18 column (YMC, 5 μm, 4.6 x 250 mm,) eluted with a 20:80 mixture of water and acetonitrile over 15 min. The flow rate was 1 mL/min, and the eluent was monitored at 254 nm using a UV detector. **1** was eluted at 10.34 min with a purity of 97.6%.

**
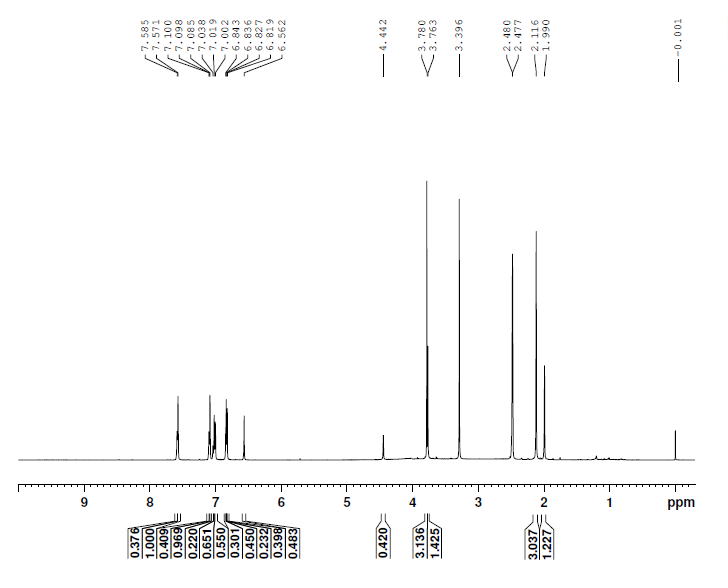
**

**Supplementary Figure 3.** **^1^H NMR spectrum of compound 1.** ^1^H NMR spectrum was recorded on a Bruker Avance 500 spectrometer at 25 ^o^C with DMSO-d_6_ as the solvent.


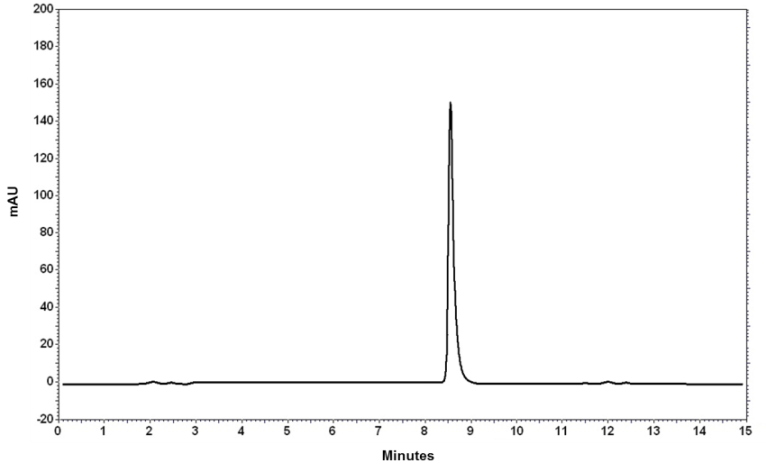


**Supplementary Figure 4. HPLC chromatogram of 2.** Compound **1** was analyzed by HPLC using a C18 column (YMC, 5 μm, 4.6 x 250 mm,) eluted with a 20:80 mixture of water and acetonitrile over 15 min. The flow rate was 1 mL/min, and the eluent was monitored at 254 nm using a UV detector. **1** was eluted at 8.63 min with a purity of 99.5%.

**
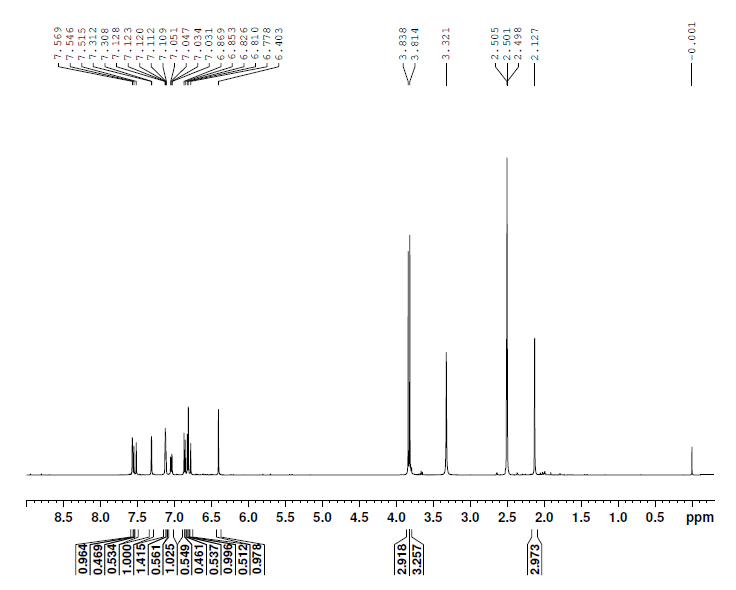
**

**Supplementary Figure 5.** **^1^H NMR spectrum of compound 2.** ^1^H NMR spectrum was recorded on a Bruker Avance 500 spectrometer at 25 ^o^C with DMSO-d_6_ as the solvent.


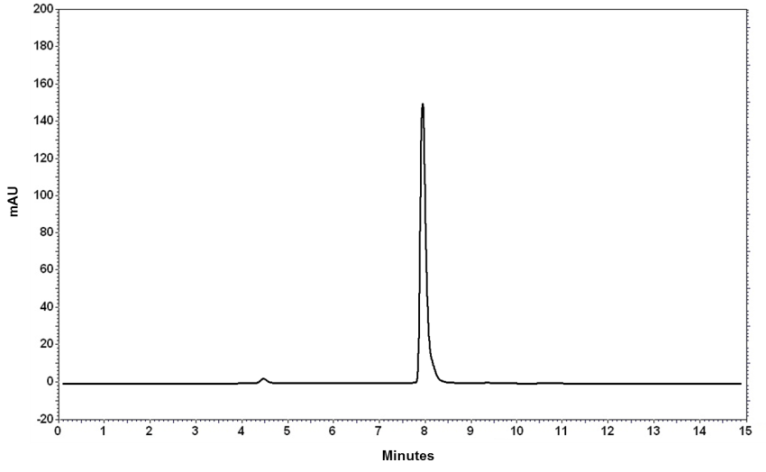


**Supplementary Figure 6. HPLC chromatogram of curcumin.** Curcumin was analyzed by HPLC using a C18 column (YMC, 5 μm, 4.6 x 250 mm,) eluted with a 20:80 mixture of water and acetonitrile over 15 min. The flow rate was 1 mL/min, and the eluent was monitored at 254 nm using a UV detector. Curcumin was eluted at 8.09 min with a purity of 98.8%.

**
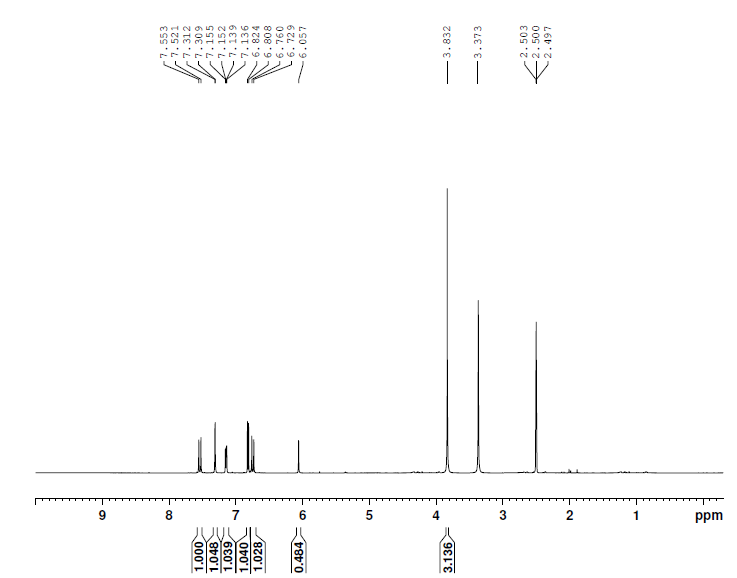
**

**Supplementary Figure 7.** **^1^H NMR spectrum of curcumin.** ^1^H NMR spectrum was recorded on a Bruker Avance 500 spectrometer at 25 ^o^C with DMSO-d_6_ as the solvent.
